# Age-Dependent Neurogenesis and Neuron Numbers within the Olfactory Bulb and Hippocampus of Homing Pigeons

**DOI:** 10.3389/fnbeh.2016.00126

**Published:** 2016-06-22

**Authors:** Virginia Meskenaite, Sven Krackow, Hans-Peter Lipp

**Affiliations:** ^1^Institute of Anatomy, University of ZurichZurich, Switzerland; ^2^The Interface Group, Institute of Physiology, University of ZurichZurich, Switzerland; ^3^Department of Physiology, School of Medical Sciences, Kwazulu-Natal UniversityDurban, South Africa; ^4^Institute of Evolutionary Medicine, University of ZurichZurich, Switzerland

**Keywords:** adult neurogenesis, birds, avian hippocampus, olfactory system, ageing, stereology

## Abstract

Many birds are supreme long-distance navigators that develop their navigational ability in the first months after fledgling but update the memorized environmental information needed for navigation also later in life. We studied the extent of juvenile and adult neurogenesis that could provide such age-related plasticity in brain regions known to mediate different mechanisms of pigeon homing: the olfactory bulb (OB), and the triangular area of the hippocampal formation (HP tr). Newly generated neurons (visualized by doublecortin, DCX) and mature neurons were counted stereologically in 35 pigeon brains ranging from 1 to 168 months of age. At the age of 1 month, both areas showed maximal proportions of DCX positive neurons, which rapidly declined during the first year of life. In the OB, the number of DCX-positive periglomerular neurons declined further over time, but the number of mature periglomerular cells appeared unchanged. In the hippocampus, the proportion of DCX-positive neurons showed a similar decline yet to a lesser extent. Remarkably, in the triangular area of the hippocampus, the oldest birds showed nearly twice the number of neurons as compared to young adult pigeons, suggesting that adult born neurons in these regions expanded the local circuitry even in aged birds. This increase might reflect navigational experience and, possibly, expanded spatial memory. On the other hand, the decrease of juvenile neurons in the aging OB without adding new circuitry might be related to the improved attachment to the loft characterizing adult and old pigeons.

## Introduction

Most avian long-distance navigators return to the site of hatching even after long periods (Bonadonna et al., 2005), suggesting that they become imprinted to the location of their birth site (Able and Bingman, 1987). The cerebral mechanisms and the interaction of the sensory processes responsible for this imprinting are still debated. There is common agreement that the process is multifactorial and shows probably species-specific variations and even within-species variation according to ecology (Beason and Wiltschko, 2015). The imprinting to a specific location is the first step in establishing a navigational map. According to Kramer’s map and compass theory (Kramer, 1953; Wiltschko and Wiltschko, 2003) this is thought to enable long-distance navigators to return to their place of hatching by comparing local parameters with those of the birthplace, using additional non-local compass mechanisms to reach the goal. Later in life, long-distance navigators learn to integrate positional mechanisms and various flight strategies to reach the goal, which may include additional targets. The mechanisms of the initial local imprinting are still debated and include olfactory cues (Wallraff, 2005; Gagliardo, 2013), local parameters of the earth’s magnetic field (Wiltschko and Wiltschko, 1996), and, possibly, gravity variations (Dornfeldt, 1991; Blaser et al., 2014), infrasound (Hagstrum, 2013) and visual information about the alignment of the horizon and the position of the sun (Köhler, 1975).

The experimental analysis of brain and sensory mechanisms has been predominantly done in homing pigeons (*Columba livia*) because they regularly return to their loft over long distances (20–1500 km). They are not natural long-distance navigators but have been selectively bred for physical and cognitive abilities to return from remote sites. Once imprinted to the coordinates of their loft, young pigeons make their first flight experience at the age of about 2 months by flying freely around the loft. Afterwards, they are trained by releasing them over increasing distances from the loft, either in peer groups or together with adults. This process involves learning of navigationally relevant cues but also the development of homing strategies and appropriate motivation. During this process, the rate of non-returning youngsters is usually much above that of adult birds, and can reach up to 50% per season, depending on geographical location. Youngsters that are not trained in their juvenile phase (mostly because of being born in late autumn) tend to be poor homers as noted by most fanciers. If the youngsters are transferred at the age of 2–3 months yet without homing training, they adapt rather easily to new locations to which they return reliably after appropriate training. This is evident from the many so-called one-loft races, in which young pigeons from many places are brought together and subjected to races, and indicates a sensitive period of navigational imprinting (Gagliardo et al., 2001). Adult pigeons having been transferred to a new loft return in most cases poorly to this site, and they occasionally re-appear at their loft of birth (Baldaccini et al., 1976). Exceptions are mobile lofts, used historically by many armies, to which the birds return after about 2 weeks of adaptation time to a new place (Blaser et al., 2013). Thus, pigeons can be conditioned to new home coordinates if they are moved with loft and partner, but the normal situation shows that pigeons return more reliably to the loft of birth after having reached their second year. Further releases increase the reliability of homing, and old homing pigeons seem to cope much better with adverse homing conditions. Obviously, this applies only to pigeons always returning, but one can expect that those aged birds had developed both, a cognitive navigational map (Blaser et al., 2013) and executive skills appropriate for homing to their loft. Formally, one can thus distinguish between a largely non-learned automated imprinting of the home loft’s geographical coordinates that remains re-programmable by transferring inexperienced youngsters to a new home loft, and a life-long accumulation of experience permitting successful homing under variable conditions in older birds. This should permit a search for neuronal correlates of imprinting, plasticity and associated lifetime changes (Barnea and Pravosudov, 2011).

Lesion or temporary inactivation studies in homing pigeons have focused primarily on the olfactory system. The main techniques included temporary inactivation of the olfactory mucosa or sectioning the olfactory nerves (Wallraff, 2005; Gagliardo, 2013), thus de-afferenting the olfactory bulb (OB). Lesion studies were targeting connected regions, namely the piriform (olfactory) cortex (Gagliardo et al., 1997) and the nidopallium caudolaterale, a homolog of the mammalian prefrontal cortex (Gagliardo and Divac, 1993). All these manipulations effectively impair homing from unfamiliar but only minimally from familiar release sites. Likewise, activation of immediate early genes (ZENK) was observed in the piriform cortex of navigating pigeons indicating functional load (Patzke et al., 2010). On the other hand, lesions of the avian hippocampus do not impair the basic ability for long-distance homing (Bingman et al., 2005) but seem to affect landmark learning and perception of navigationally relevant cues during flight (Gagliardo et al., 2014; Herold et al., 2015).

Since the age of homing pigeons is usually well documented by means of breeder records and foot rings, it is possible to undertake cross-sectional studies aimed at detecting age-dependent changes in neuronal plasticity in those structures. Postnatal and adult neurogenesis has become a prominent marker for identifying plasticity processes in particular brain regions of mammals and also birds. In mammals, adult hippocampal neurogenesis by progenitor granule cells in the dentate gyrus peaks at juvenile periods and decreases strongly thereafter (Amrein and Lipp, 2009; Ben Abdallah et al., 2010; Amrein et al., 2011). A similar time course is observed in the olfactory system although adult neurogenesis there (evident by migrating cells to the OB) is declining less rapidly.

In birds, adult neurogenesis is more widespread than in mammals, new cells being generated in the ventricular zone appearing not only in the medial (parolfactory lobe) and lateral striatum, the OB and the hippocampal complex but also in a variety of forebrain structures such as the hyperpallium and nidopallium caudolaterale, the dorso-lateral corticoid and various song control nuclei (Vellema et al., 2010; Barnea and Pravosudov, 2011). Partially, the distribution of newly formed neurons in the brain of birds appears to mark species-specific specialized regions with a high degree of neuronal plasticity, but there seem to be other developmental and genetic mechanisms directing migration (Vellema et al., 2010). In terms of avian navigation, the few available studies suggest a link between neuronal recruitment in hippocampus with migratory distance, long distance migrants showing higher levels (LaDage et al., 2011; Barkan et al., 2014, 2016).

Age-dependent differences in adult neurogenesis have been observed sporadically in the canary brain (Alvarez-Buylla et al., 1994; Wilbrecht and Kirn, 2004), in zebra finches (DeWulf and Bottjer, 2002; Wang et al., 2002; Pytte et al., 2007) and ring doves (Ling et al., 1997). However, there seems to be no systematic study assessing the time course of adult neurogenesis throughout the lifespan of birds. Therefore, we set out to document the level of neurogenesis in the OB and the hippocampal formation of homing pigeons covering juvenile, adult and aging periods, counting also whether there were age-related changes in total neuronal number in these regions.

We focused on determining the numbers of newly-born vs. mature periglomerular neurons in the OB, since doublecortin (DCX) immunoreactivity is very sparse and weak in the granule cell layer of the OB in pigeon, and our earlier trials with BrdU injections (unpublished data) labeled much larger numbers of periglomerular cells than granule cells.

## Materials and Methods

This study was carried out under the license 28/2012 of the Veterinary Office of the Canton of Zürich in accordance with the Swiss regulations for use of experimental animals.

### Pigeons

The brains of 35 pigeons ranging in age from 1 to 168 months were investigated. Young and young adult pigeons were obtained from the loft of one of the authors in Switzerland (H-PL), while older and aged birds were purchased from breeders in Switzerland, Italy and Germany. The latter were probably not the best racing pigeons and have thus no record of performance, but had returned reliably from races or training releases during several years while other birds from the same loft were lost over time. Sex was determined by inspecting gonads at the end of dissection. In juvenile 1–2 month-old birds, sex was not determined due to immature glands. Therefore, an analysis of potential sex differences did not seem meaningful. To minimize seasonal variations on neurogenesis and anatomical size, all pigeons were sacrificed during a narrow annual time-window from mid-November to mid-December.

### Histology

Each pigeon was deeply anesthetized with pentobarbital (200 mg/kg) and perfused transcardially with a fixative consisting of 4% paraformaldehyde in 0.1 M phosphate buffer pH 7.4. Brain weight was determined in 25 birds, one age class (80 months) had no information because of lost records. All brains were postfixed for 4 h in the same fixative and sectioned at midline into left and right parts. One hemisphere was dehydrated, embedded into Technovit-resin, and serially sectioned through at 20 μm. The obtained sections were stained with Giemsa and used to quantify the general number of neurons and volumes of areas of interest.

The other hemisphere was equilibrated in 10–30% sucrose and serially cryosectioned in the coronal plane. Forty-micron-thick sections were collected in eight series and stored in cryoprotecting solution at −20°C. Regularly spaced sections (320 μm) were used for immunohistochemistry of DCX. Whenever possible, samples from different birds and age classes were processed simultaneously to avoid batch-dependent differences in intensity of immunostaining. For immunostaining, sections were pretreated for 20 min with 1% hydrogen peroxide. Then they were preincubated in 10% normal rabbit serum in tris-buffered saline, containing 0.2% Triton X-100, and incubated for 36 h in goat polyclonal antibody to DCX. The secondary antibody, biotinylated rabbit anti-goat IgG (1:250; Vector Labs), and Vectastain ABC Elite reagent (1:100; Vector Labs) were applied for 2 and 1 h, respectively. Standard 3,3’-diaminobenzidine reaction was used to visualize DCX positive cells. Sections were mounted onto slides, air dried, and coverslipped without counterstaining. Since the proliferation marker most commonly used in mammals, Ki67, works poorly in birds, we also immunostained corresponding sections for the proliferation marker proliferating cell nuclear antigen (PCNA) and for a second marker for young neurons (PSA-NCAM). However, the results for these two markers were extremely variable, indicating technical problems in the adaptation of the staining procedure. Therefore, we present only the data for Giemsa and DCX stains, characterized by reasonable inter-individual variability of staining.

### Morphometry

**Figure 1:**
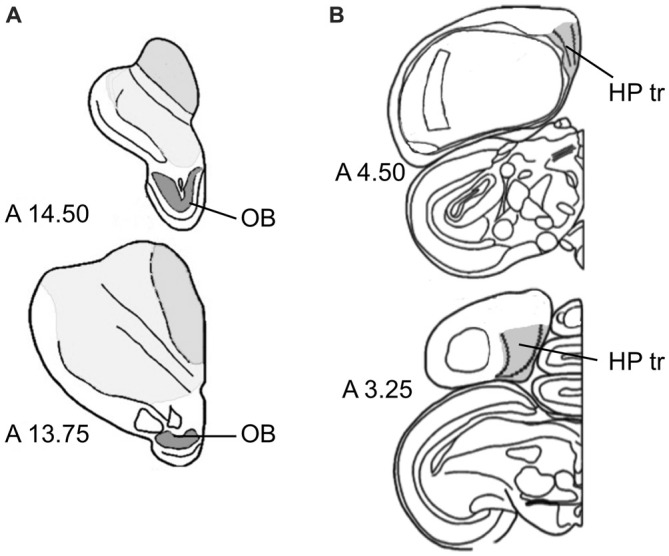
**Sampling regions. (A)** Olfactory bulb (OB) at two antero-posterior planes of the atlas of Karten and Hodos (1967). **(B)** Triangular area of the hippocampal formation (HP tr) on two antero-posterior planes (Karten and Hodos, 1967), exact delineations can be found in Atoji and Wild (2004) and Herold et al. (2015).

Within the OB, DCX-positive cells and Giemsa-stained neurons were quantified in the periglomerular cell layer. Volume measurements included the entire OB (Figure 1A). The triangular area of the hippocampus (HP tr), which we used for sampling in this study, was delineated according to the hippocampal boundaries of Karten and Hodos (1967), and corresponding to the trilaminar complex (lateral and medial layers with triangular part between them) of the hippocampal formation by Atoji and Wild (2004) within the V-shaped structure in the medial wall (Figure 1B), see also Herold et al. (2015). For unstained sections used for counting DCX-positive neurons, boundaries of the regions of interest were determined by nuclear staining of alternate sections from the same hemisphere.

All quantifications were performed using unbiased stereological quantification on Zeiss Imager M2, equipped with a high-resolution video camera CX 900 (mbf Bioscience) and the software package StereoInvestigator 9.0–10 (mbf Bioscience).

The number of adult (Giemsa-stained) neurons in the periglomerular layer of the OB was sampled using an optical fractionator from every 6th section (120 μm interval), which resulted on average in 13 sections per single bulb and coefficients of error (Gundersen) *m* = 0, mean ± SD, 0.07 ± 0.01; *m* = 1, 0.06 ± 0.01. The neuron number in the HP tr was sampled from every 24th section (480 μm), on average 11.5 sections per HP, *m* = 0, 0.08 ± 0.01; *m* = 1, 0.07 ± 0.01. In the Giemsa-stained sections, neurons were differentiated from glial cells according to their generally larger size, the bigger diameter and shape of proximal processes, and a darker, violet-blue shade of nucleus and cytoplasm, which in glial cells appear sky-blue. The neuron identification criteria were initially confirmed on NeuN immuno-reacted, dehydrated and Giemsa-counterstained sections.

The numbers of DCX positive cells were estimated using an optical fractionator on every 8th (320 μm) or every 16th (640 μm) section for OB and HP tr, respectively. This scheme resulted in measuring 4–5 sections, *m* = 0, 0.165 ± 0.03, *m* = 1, 0.14 ± 0.03 for OB, and 9–10 sections, *m* = 0, 0.14 ± 0.03, *m* = 1, 0.13 ± 0.03 for HP tr.

The volumes for the whole OB and HP tr were measured using Cavalieri estimator; every 6th section, *m* = 0, 0.03 ± 0.01, *m* = 1, 0.02 ± 0.00 for OB, and every 18th section (360 μm; 16 sections measured), *m* = 0, 0.03 ± 0.01, *m* = 1, 0.02 ± 0.00 for HP tr.

### Statistics

Neuron numbers in the regions of interest were linearly regressed on age (Bates et al., 2015), as no quadratic component appeared to be of significance (*p* > 0.5 in all cases). The effect of age and neuron number on volume were determined in structural (causal) models (Rosseel, 2012), accommodating the linear coefficients of age on neuron number and volume, and of neuron number on volume.

The effect of age on number of DCX stained cells and %DCX (number of DCX positive cells divided by total cell number) exhibited a significant quadratic term for both regions (*p* < 0.05 in all cases) and boxcox transformation yielded maximum log-likelihood estimates of λ near 0, hence DCX cell numbers and %DCX were log-transformed for analyses. Since correlations of DCX cell number with neuron numbers, partialled for age, were non-significant (*p* > 0.3 in both cases), neuron number was not entered as covariate in models for DCX stained cells.

Effects were considered significant at *p* < 0.05 throughout. Data were analyzed in R (R Development Core Team, 2015) and plotted using ggplot2 (Wickham, 2009).

## Results

Table 1 summarizes findings, and shows brain weights that reach adult levels at the approximate age of 6 months.

**Table 1:** **Cell counts and brain weight (Mean ± SEM)**.

Age class	*n*	g bodyweight	g brain weight	Neurons HP tr	Neurons OB pgl	DCX HP tr	DCX OB pgl	Vol HP tr mm^3^	Vol OB mm^3^
1 month	6	355 ± 22	1.65 ± 0.06	739,313 ± 38,768	47,642 ± 2263	20,513 ± 1359	1039 ± 121	7.972 ± 0.206	2.224 ± 0.396
2 months	5	438 ± 12	1.89 ± 0.03	772,745 ± 27,148	59,515 ± 7338	19,845 ± 3502	862 ± 192	8.258 ± 0.235	2.566 ± 0.161
6 months	5	433 ± 15	2.06 ± 0.04	798,036 ± 61,863	57,801 ± 2896	16,650 ± 3330	643 ± 97	8.470 ± 0.253	3.011 ± 0.156
12 months	6	475 ± 21	2.17 ± 0.07	975,437 ± 64,558	35,609 ± 2917	13,350 ± 2903	416 ± 115	8.990 ± 0.380	2.614 ± 0.138
2–3 years	4	469 ± 26	2.09 ± 0.07	921,604 ± 46,647	50,918 ± 6717	13,444 ± 3604	218 ± 97	8.747 ± 0.318	2.392 ± 0.178
6–7 years	6			1,354,974 ± 86,519	34,870 ± 4598	10,688 ± 1110	151 ± 25	9.785 ± 0.379	2.737 ± 0.150
12–14 years	3	482 ± 13	2.19 ± 0.07	1,682,386 ± 138,693	36,879 ± 2844	11,050 ± 1537	53 ± 26	9.920 ± 0.578	2.666 ± 0.190

*HP tr, Hippocampal triangular area; OB, OfactoryBbulb; DCX, Doublecortin positive neurons; pgl, Periglomerular layer*.

### Hippocampal Formation

#### Cell Numbers

**Figure 2:**
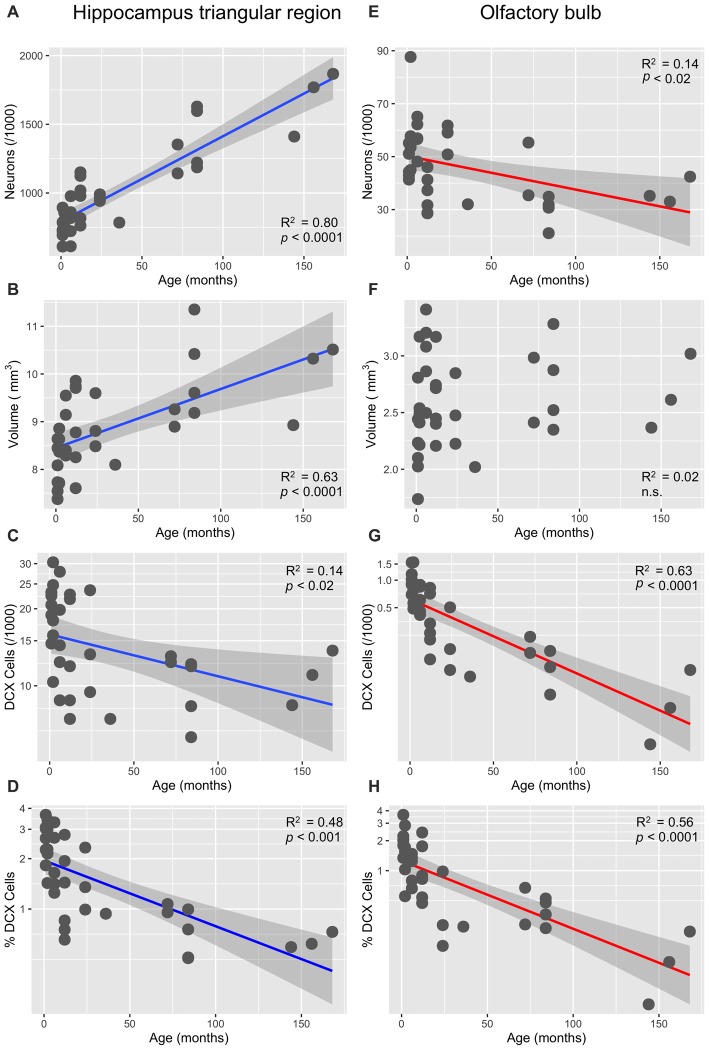
**Age-dependency of neuronal numbers and volumes in the pigeon’s triangular region of the hippocampal formation and in the OB (linear slopes with 95% CI). (A)** Strong increase of mature neurons in the triangular region (HP tr). **(B)** Concomitant increase of the volume of HP; **(C)** significant decrease of doublecortin (DCX)-positive cells in HP tr, log-transformed cell numbers. **(D)** Significant age-dependent decrease of the percentage of DCX-positive cells (of total cell number) in the HP tr, log-transformed percentages (see also Figure 4A). **(E)** Significant decrease of neuron number in the periglomerular layer of the OB. **(F)** Huge variability of volume of the OB in young pigeons but no significant decrease with age. **(G)** Massive decrease of DCX-positive neurons in the periglomerular layer of the OB, log-transformed cell numbers. **(H)** Same decrease expressed as percentages of total neurons in the periglomerular layer, log-transformed (for better separation of age levels see also Figure 4B).

**Figure 3:**
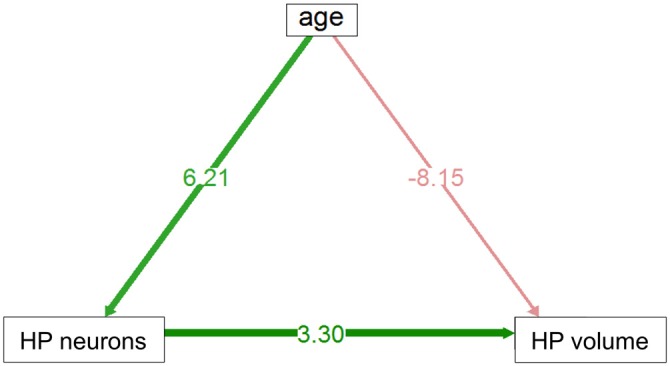
**Direct and indirect effect via neuron number of age volume in HP tr.** Positive indirect effect is highly significant, negative direct influence is of marginal significance (see text).

In the HP tr formation, we observed a massive increase in cell numbers across age levels. From an average level of about 740,000 neurons in 1-month old birds, the numbers appeared doubled in old adult birds and peaked in the oldest pigeon (14 years) at 1.9 million cells (Table 1, Figure 2A). This increase was paralleled by a significant increase in volume of the HP tr (Figure 2B), which appeared to be less pronounced in the very old birds, indicating an increased neuronal density in that age group. Statistically, neuron number increased by 6200 cells per month of age (*F*_(1,33)_ = 134.89, *p* < 0.001, adj. *R*^2^ = 0.80). The causal model (Figure 3) revealed that hippocampal neuron number affected volume positively (3.3 × 10^3^ μm^3^ per neuron), so the positive indirect effect of age on volume (20.5 × 10^3^, *z* = 4.66, *p* < 0.0001) was much stronger than the negative direct effect (−8.2 × 10^3^, *z* = −1.81, *p* < 0.071). Additional information about cell numbers in the early phases of life (months 1–12) is provided in Table 1.

#### Neurogenesis

**Figure 4:**
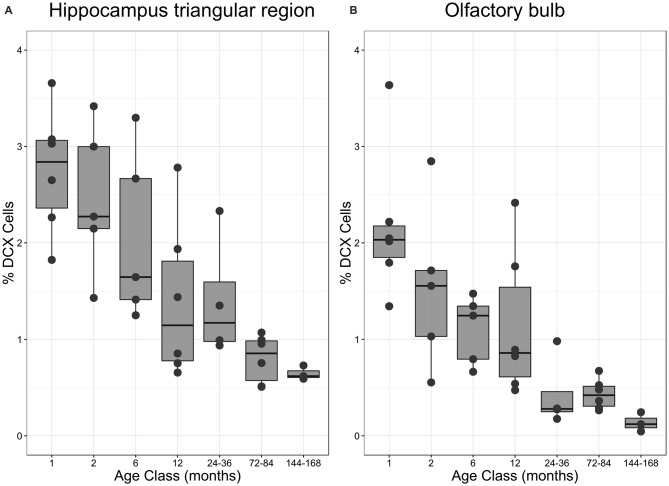
**Proportion of DCX-positive neurons in different age classes. (A)** Percentage of DCX-positive neurons in the triangular area of the hippocampal formation (HP tr) as related to the number of mature neurons in the HP tr. **(B)** Percentage of DCX-positive neurons in the periglomerular layer of the OB as related to the number of mature neurons in the same structure. Boxes expose median and interquartile, whisker and dots indicate total data range. For statistics see text and for regressions Figures 2D,H.

The number of newly born DCX-positive neurons in the HP tr declined from about 20000 in 1-month old pigeons to 13000 cells in 12-month old birds, leveling off to about 11,000 cells in the oldest birds (Table 1). Statistically, DCX stained cell number decreased significantly with age (*F*_(1,33)_ = 6.63, *p* < 0.02, adj. *R*^2^ = 0.14), most of the variation occurring in the first year of life (Figure 2C). For comparison with other species, we show the changes in form of percentages of the total neuron number in the HP tr formation by age class (Figure 4A). The regression of log %DCX on age (Figure 2D) indicates a decrease of young neurons from an initial level of 1.6–2.3% to a final level of 0.27–0.68% (*F*_(1,33)_ = 32.27, *p* < 0.001, adj. *R*^2^ = 0.48), see also Figure 4A). However, low numbers of DCX-positive cells could be found in several pigeons already at 12 months or younger (Figure 2C).

### Olfactory Bulb

#### Cell Numbers

In the periglomerular layer, cell numbers are high during the first 6 months but show large variability, however (range 40000–90000 cells, Table 1). After this period, individually different declining of OB cell numbers was observed. Young adult pigeons (12 months old) showed nearly the same number of neurons as the old adult and aged pigeons showing on average the lowest numbers. Overall, there was a weak yet statistically significant decrease of 130 periglomerular cells per month of age (*F*_(1,33)_ = 6.63, *p* < 0.02, adj. *R*^2^ = 0.14, Figure 2E). The volume of the entire OB was highly variable in young pigeons but appeared independent from age (Figure 2F, Table 1). Since neuronal cell counts were not done for the granule cell layer, a causal analysis including age effects on both cell numbers and volumes was not done.

#### Neurogenesis

The number of DCX-positive cells in the periglomerular layer of the OB decreased highly significantly with age (*F*_(1,33)_ = 59.82, *p* < 0.001, adj. *R*^2^ = 0.63, Figure 2G). The numbers were extremely variable during the first 6 months (Table 1). Overall, DCX-positive cells declined from an initial level of about 1000 neurons at 1 month to levels between 50–150 cells in adult, old adults and aged pigeons, in which the variability appeared much lower (Figure 2G, Table 1). When expressed as percentages of the total periglomerular cell number in the OB, regression of log %DCX on age (Figure 2H) revealed a 10-fold decline from 0.95 to 1.63% DCX-positive neurons in 1-month old pigeons, to about 0.05–0.18% in the oldest bird (*F*_(1,33)_ = 45.03, *p* < 0.001, adj. *R*^2^ = 0.56). For details in the age groups see also Figure 4B.

## Discussion

Our data show that the number of neurons in the triangular region of the pigeon hippocampus increased gradually in the young birds but peaked remarkably in the oldest pigeons. Interestingly, the number of DCX-positive neurons decreased only by a factor 2 over the lifespan. Conversely, the number of mature neurons in the periglomerular layer of the OB decreased moderately over the lifespan of the pigeons, while the number of DCX-positive neurons fell strongly after 6 months, and persisted at low yet variable levels through the rest of the life of the investigated pigeons.

### General Findings

Assuming that DCX is a reasonable proxy for postnatal neurogenesis in birds (Balthazart et al., 2008), we can thus conclude that the lifetime course of adult neurogenesis in homing pigeons follows somewhat similar pattern observed in many mammalian species (Amrein et al., 2011), namely an early peak followed by a rapid decline and eventual leveling off at low rates (Ben Abdallah et al., 2010). However, the levels of hippocampal neurogenesis during the first year differ between species. At 1 month of age, young mice showed about 5% of DCX-positive cells among mature granule cells, decreasing to 0.25% at 9 months. In the pigeons, initial levels of DCX-positive cells were around 3% at 1 month but still 1.5% at the age of 12 months. Thus, in a comparable time span, there appears a reduction by a factor 20 in mice but in pigeons by a factor 2 only, indicating a much higher long-lasting level of adult neurogenesis adding to the overall hippocampal cell population. Given the unreliable results of PCNA staining, we cannot determine the relation between true proliferation and DCX-staining, but at least in some old birds we observed PCNA positive cells, indicating that proliferation was also going on. Much in contrast to mammals, however, the cell numbers in the pigeon hippocampal formation increased massively. Given the concomitant decline of adult neurogenesis in older birds, this strongly implies that new neurons were added and were not simply replacing older cells. Why the OB showed a moderate decrease in neuronal number associated with a strong drop in adult neurogenesis is not clear at present. Since the sample was heterogeneous with respect to origin and life experience of the older birds, more data are needed to reach firm conclusions about the loss of neurons in the OB. The observed decrease in neurogenesis, however, is so strong that a chance effect is unlikely.

### Functional Implications

A functional interpretation of the findings remains speculative yet, because in such a retrospective study, it is impossible to disentangle the various components of lifetime experience including navigation, and the effects of selection effects on the brains of the birds as compared to those not returning to the loft.

### Avian Hippocampus

The age-dependent increase of mature hippocampal neurons in the pigeon brain fits explanations associating the volume or cell number of the hippocampus with superior navigational capacities or even expanded spatial memory of migratory birds (Healy et al., 1996; Cristol et al., 2003; Mettke-Hofmann and Gwinner, 2003; Pravosudov et al., 2006), even though homing behavior of Junco migrants and non-migrants with different hippocampal volumes was equal (Keiser et al., 2005). In the same vein, it has been found that acute navigational training increases the volume of the adult avian hippocampus moderately (Cnotka et al., 2008), although it is not known whether such an increase persists. For a review of the role of the pigeon hippocampal formation in pigeon navigation, see Herold et al. (2015). Importantly our old birds with increased hippocampal neuron number were survivors, as homing pigeons disappear gradually from a loft when subjected to races or release experiments. Reliable homing is only partially dependent on superior navigational abilities: it also includes attachment to the loft, motivation, physical shape, executive functions, social position, behavior in facing raptors and many more aspects (Lipp, 1996; Barnea and Pravosudov, 2011). Therefore, a safe conclusion is that the age-dependent increase of the hippocampal formation reflects lifetime experience of survivors, and, possibly, a role of the avian hippocampus in orchestrating different neuronal networks covering executive, cognitive and sensory functions.

A positive effect of hippocampal neurogenesis on navigational capacities appears to be indicated by more recent reports about differences in hippocampal neurogenesis in non-migrating and migrating species, usually reporting higher rates in migrants (Barkan et al., 2014, 2016; Barnea and Pravosudov, 2011; Herold et al., 2015; LaDage et al., 2011). These reports do not address the phenomenon of age-dependent decline, which paradoxically would seem detrimental for older and thus successfully migrating birds. Linking decreasing hippocampal neurogenesis with growing navigational experience is thus conceptually difficult at present. Mammalian long-distance navigators and migrants such as bats and flying foxes have low or missing rates of adult hippocampal neurogenesis (Amrein et al., 2007; Gatome et al., 2010), and the same has been reported for minke whales (Patzke et al., 2015). One hypothesis explaining the role of growing experience with concomitant decrease of adult hippocampal neurogenesis proposes that new neurons in elderly brains might serve other functions than required in the juvenile period (Couillard-Despres et al., 2011; Couillard-Després, 2013). Another view combining the role of decreasing adult neurogenesis in both structures is given below.

### Changes in the Olfactory Bulb

Given the important role of the olfactory system in pigeon navigation, the observed decrease of mature periglomerular olfactory neurons was somewhat unexpected. In view of the heterogenic sample size, larger samples are probably needed to verify the findings, but an increase such as in the hippocampal formation seems unlikely. Likewise, the rapid decline of olfactory neurogenesis in young birds is most certainly not a chance event. Moreover, this decrease fits the navigational ontogeny of pigeons. It appears that high levels of OB neurogenesis reflect a sensitive period of navigational imprinting to the home loft coordinates (Gagliardo et al., 2001), but permit also easy adaptation to new places when youngsters up to 6 months of age are transferred to other lofts. Whether learning of new loft coordinates as observed with mobile lofts reflects the presence of ongoing adult neurogenesis, albeit at low levels, remains to be studied. In any case, low levels of OB neurogenesis seem to be confined to older and reliably homing birds. For such birds, navigational flexibility with respect to new loft positions is no longer necessary. Thus, as hypothesized for mammals, the seemingly paradoxical decline of adult neurogenesis in individuals attaining reproductive competence may reflect stabilization and permanence of acquired routines at the expense of juvenile flexibility (Amrein and Lipp, 2009). Low levels of adult neurogenesis in the OB adult birds may thus relate to solid anchoring of a relevant navigational target, while the reduction of hippocampal neurogenesis may reflect entrenchment of both, successful navigation strategies and appropriate coping with recurring environmental situations necessary for the survival of a homing pigeon.

## Conclusion

- The lifetime course of adult neurogenesis in two structures of the pigeon brain follows a general rule observed in mammals: juvenile peaking followed by a logarithmic decline when entering adult age, and subsequent persistence at lower levels that appear, however, much higher than in rodents.
- Adult neurogenesis increases permanently the number of mature neurons in the pigeon HP tr, possibly reflecting accumulated experience necessary for reproduction, survival and navigation.
- The decay of adult neurogenesis in the OB may correspond to the end of a sensitive period for imprinting positional information of the home loft.

## Author Contributions

VM initiated and carried out the study, H-PL managed logistics and support. Data analysis was done by SK. The manuscript was written by H-PL with contributions from VM and SK.

## Conflict of Interest Statement

The authors declare that the research was conducted in the absence of any commercial or financial relationships that could be construed as a potential conflict of interest.
